# A systematic review and meta‐analysis examining the impact of placement instability on the mental health outcomes of care experienced children and young people

**DOI:** 10.1002/jcv2.70099

**Published:** 2026-01-31

**Authors:** Rosa Sparks, Gary Kainth, Helen Minnis, Jala Rizeq

**Affiliations:** ^1^ School of Health and Wellbeing University of Glasgow Glasgow UK; ^2^ Gillberg Neuropsychiatry Centre University of Gothenburg Gothenburg Sweden

**Keywords:** care experienced children and young people, mental health, meta‐analysis, placement instability

## Abstract

**Background:**

Care experienced children and young people (CECYP) are at risk of mental health difficulties. This review aimed to examine the impact of placement instability on the mental health outcomes of CECYP and to explore how placement instability is measured.

**Methods:**

This review was conducted following the PRISMA guidance. Four databases (PsycINFO, Embase, Medline and ProQuest) were initially searched on 14^th^ December 2023, with an updated search conducted on January 29^th^ 2025. The following inclusion criteria were used: quantitative observational studies, CECYP (0–18 years), mental health difficulties as the outcome and placement instability as the exposure. The quality of the included studies was also assessed. The results from all papers included in the review were narratively synthesised and results from a subsample of the papers eligible for quantitative synthesis were analysed using a random effects meta‐analysis to examine the association between placement instability and mental health outcomes of CECYP.

**Results:**

Twenty‐two studies were eligible for inclusion. The measurement of placement instability varied with some studies counting number of placement moves and some grouping number of moves into levels. The time frames in which moves were measured also varied. Overall, placement instability had a negative impact on mental health outcomes in CECYP, irrespective of age, sex, domain of mental health assessed (internalising and externalising), and initial levels of mental health. The meta‐analysis found that placement instability had a small significant association with both internalising (*r =* 0.14, 95% CI = 0.12, 0.17) and externalising (*r =* 0.14, 95% CI = 0.11, 0.18) mental health difficulties.

**Conclusion:**

Placement instability is inconsistently measured and defined in the literature. Despite this heterogeneity in operationalisation, it remains a concerning risk factor for mental health difficulties of CECYP. Efforts should be made to minimise instability for this population.

**Trial Registration:**

The protocol was registered on PROSPERO on the 2nd of February 2024 (CRD42024444031), can be found at https://www.crd.york.ac.uk/PROSPERO/view/CRD42024444031.

## Key points

**What's known?**

- Care experienced children and young people (CECYP) experience high placement instability and are at risk for negative health outcomes.

**What's new?**

- In this systematic review and meta‐analysis, we found a small but robust significant association between placement instability and both internalising and externalising difficulties in CECYP. In many cases this effect was irrespective of age and sex and in some cases above and beyond initial levels of mental health difficulties.

**What's relevant?**

- Policy should be put in place to ensure stability in caring placements.
- Interventions are needed to support carers in managing the mental health needs of CECYP to prevent a negative cycle of instability and mental health difficulties.

## INTRODUCTION

Children enter the care system for various reasons, most commonly due to maltreatment including physical, emotional and sexual abuse, and also due to exposure in the home or community to alcohol, drug misuse or violence (NICE, 2021). Early maltreatment can be a risk factor for the development of mental health difficulties, both internalising and externalising difficulties (Lippard & Nemeroff, 2020; Muniz et al., 2019). Internalising difficulties are characterised by problems with worry, mood, anxiety and withdrawal, whereas externalising difficulties represent observable behaviours such as impulsivity, aggression, and hyperactivity (Achenbach, 1966). Care experienced children and young people (CECYP) are three times more likely to have a diagnosis of a mental health disorder than children who have not been in care (Lohr & Jones, 2016). Some of the most common diagnoses of CECYP across the internalising and externalising domains include behavioural disorders, major depressive disorder, post‐traumatic stress disorder, and reactive attachment disorder (Cummings & Shelton, 2024; Engler et al., 2022). It has been suggested that these difficulties could be a risk factor for further placement breakdown (Engler et al., 2022). The journey in care for many children is characterised by instability in placements, causing disruption to relationships and schooling and possibly exacerbating mental health difficulties (Woodall et al., 2023). Therefore, it is important to understand the extent to which placement disruptions or moves have an impact on CECYP's mental health.

Care experience is defined as CYP who are looked after away from home, for any length of time and could include placements in a residential children's house, in a foster placement or in a kinship placement and also includes CYP who have been adopted (Scottish Government, 2024). Estimates suggest that a third of youth in foster care experience three or more placement moves during their time in care (Rubin et al., 2004). Placement instability is defined as ‘household and/or institutional moves or placement changes that do not result in a child's permanent placement’ (Fisher et al., 2013). A 2019 meta‐analysis found that child age (younger), placement without siblings and history of maltreatment as important predictors of placement instability, with child behavioural difficulties noted as the strongest predictor (Konijn et al., 2019).

Previous research has suggested that behaviour problems might be both a cause and a consequence of placement instability (Newton et al., 2000). Although the evidence base on the predictors of placement instability has been extensively studied, the effect of placement instability on mental health problems, including both internalising and externalising difficulties requires further consideration (Engler et al., 2022). A recent study by Maguire et al. (2024) reviewed quantitative and qualitative studies on the effect of placement instability on emotional and behavioural outcomes of children in foster care, concluding that placement instability has a negative impact on both domains. We are yet to examine and synthesise the evidence base on the effect of placement instability on the mental health of CECYP, in any placement type.

### The current review

The purpose of this review was to synthesise, quantify, and examine the quality of the quantitative evidence base regarding the impact of placement instability on the mental health outcomes of CECYP. It is hypothesised that CECYP who experience high placement instability will have worse mental health outcomes than those with low instability. The primary question of this review was:

1. What is the association between placement instability and mental health outcomes of CECYP?

The secondary question of the review was:

2. How is placement instability measured in studies examining the impact on mental health outcomes of CECYP?

## METHODS

### Protocol and registration

This review was conducted in line with the updated Preferred Reporting Items for Systematic Reviews and Meta‐analyses (PRISMA) guidelines (Page et al., 2021). The protocol was registered on PROSPERO on the 2^nd^ of February 2024 (CRD42024444031).

### Eligibility criteria

All papers included in the review were written in English language and published in peer‐reviewed journals. The full eligibility criteria can be found in Table 1.

**TABLE 1:** Eligibility criteria.

Inclusion	Exclusion
English language papers	Dissertation abstracts or papers published in non‐peer‐reviewed journals
Published in peer reviewed journals	Papers written in non‐English languages
Quantitative observational studies (including cross sectional and longitudinal).	Randomised controlled trials (RCTs) or non‐observational designs
Population included children aged between 0 and 18 years. This age range was used as most representative of children from birth to completion of secondary school, and still under the care of carers or parents. This is also the age range used by the World Health Organization for children (2020) as those aged 0–18 years.	Papers that measure the effect of mental health difficulties as the exposure on placement instability as the outcome
All children and young people must have experienced time in care, including any care type (foster, kinship, institutional and adoption).	
Measuring a mental health outcome, using a mental health difficulties outcome scale (total mental health difficulties or internalising or externalising mental health difficulties).	
Measuring the association between placement instability (the exposure) and the above outcome.	

### Search

Search strategy was developed in consultation with an NHS and University of Glasgow librarian. Studies were identified through searches of four major electronic databases: PsycINFO, Embase, Medline and ProQuest. There were no restrictions on publication date. A search was undertaken, with the following key words: (foster family or care or parent* or carer* or home or child* or youth or young) or (kinship care or relative care or out‐of‐home‐care or out of home care or looked after or looked‐after) and (placement* instability or stability or move* or change* or number*). The keywords were selected by reviewing previously published studies and reviews on similar topic areas.

### Study selection

Duplicates were removed using the automatic function on EndNote and additionally reviewed by hand. The primary researcher (RS) screened 100% of titles and abstracts, using the inclusion and exclusion criteria. A second doctoral trainee assisting with the research screened 10% of titles and abstracts to assess for inter‐rater agreement, and found an 97% agreement. Discrepancies between reviewers were resolved by discussion with input from senior author (JR). Using eligibility criteria, full text screening was completed for the remaining papers, with 100% of the full text papers screened by the primary author (RS). The second reviewer screened 10% of the full text papers to assess for inter‐rater agreement and found an 100% agreement. Reason for exclusion at this stage was recorded. Following this, reference lists in recent and relevant reviews on the topic were hand searched for relevant papers (Engler et al., 2022; Konijn et al., 2019; Maguire et al., 2024).

### Data extraction and coding of study characteristics

A data extraction table was created for this study. All information was extracted by the primary author (RS). All relevant statistics extracted were independently reviewed for accuracy by senior author (JR). The following information was extracted from the included papers:

a) Key study characteristics: author, publication year of study, country where study was conducted, study design.
b) Sample characteristics: total sample size, child age, sex.
c) Exposure: measure of placement instability or moves, and how these were measured as well as the time frame in which placement moves were measured.
d) Mental health outcomes: name of mental health measure used and scoring (e.g., continuous score or binary clinical cutoff).
e) Analysis and effect: number of subjects in the analysis, statistic and effect size, *p* value, standard error, confidence interval and covariates where reported.

### Quality of individual studies and risk of bias

Study quality was assessed using the Crowe Critical Appraisal Tool (CCAT) (Crowe, 2013), which has also been used as an appraisal tool by other systematic reviews in the field of child mental health (Mercier & Dorris, 2024; Walker et al., 2024). The CCAT form is divided into eight categories (preliminaries, introduction, design, sampling, data collection, ethical matters, results and discussion) with 22 items. Each category receives its own score on a six‐point scale from 0 to 5, with zero indicating the lowest score and five indicating the highest score. The total score given to a paper is the sum of all the categories, and this can be expressed as a percentage. The total % CCAT quality scores are classified as:< 20 (50%) poor quality; 20–30 (50%–75%) moderate quality and 30+ (75% +) high‐quality (Crowe, 2013). A second rater completed 10% of the critical appraisal using CCAT and inter‐rater agreement was calculated, with an 100% agreement on ratings of the overall quality of the studies, and no more than a one‐point difference in ratings across all categories. These were conferenced and a consensus was reached.

### Synthesis

To examine how placement instability or moves are measured, a descriptive approach was used. To assess the effect of placement instability on mental health outcomes (primary research question), initially a narrative synthesis was utilised to integrate and summarise findings across all included studies. The approach taken included developing preliminary exploration and synthesis, examining relationships within and between studies, and assessing the quality of the studies included (Popay et al., 2006).

A meta‐analysis was also conducted with a subset of eligible studies. The R package metafor (Viechtbauer, 2010) was used to conduct a meta‐analysis of correlations in RStudio (R version 4.33, R Core Team, 2024). Due to expected heterogeneity across studies, meaning differences in study design and measurement, a random‐effects model was used with a restricted maximum‐likelihood estimator (Vevea & Coburn, 2015). In metafor, as is common in meta‐analyses of correlations, in order to meta‐analyse the correlations, we had to initially standardise those effects using Fisher's *z* transformation. Those effects were then transformed back to *r* correlations to aid with interpretation. The study specific and pooled correlations and combined correlation along with their 95% confidence interval are visualised using a forest plot. Cohen's criteria (Cohen, 1988) for small (*r* = 0.10–0.29), medium (*r* = 0.30–0.49) and large (*r* ≥ 0.50) effect sizes were used to evaluate the magnitude of the effect sizes. Heterogeneity among studies was analysed using the Cochrane's *Q* test (significant heterogeneity was determined at p < 0.05). The I^2^ was also used to characterise heterogeneity as small = 25%, moderate = 50%, and large = 75% (Higgins et al., 2003). To detect outliers that might have an effect on the results when large heterogeneity is indicated, we used the following approach: we removed a study when the confidence interval of that study does not overlap with the confidence interval of the pooled effect (Harrer et al., 2021). When an outlier was detected, the meta‐analysis was rerun with that study removed.

Effect size. The effect size of each study was extracted and recorded individually. An online effect size converter was used to convert odds ratio and standardised regression coefficients to *r* (Lenhard & Lenhard, 2022). Effect sizes were documented for internalising, externalising, and total difficulties separately. Where various effect sizes within a domain were reported, or when multiple effects were reported for the same domain using multiple informants, an average was taken. This was considered the most reasonable approach to retaining data and ensuring that the effects used are most representative of the mental health outcome in question. Overall, two studies reported separate results by multiple informants. Four studies reported on multiple effects across multiple domains of externalising/internalising problems that were averaged to be most consistent conceptually as we were interested in overarching externalizing and internalizing domains. When longitudinal data reported on multiple follow ups, the effect of the exposure on the outcome at the latest follow up was included.

## RESULTS

**FIGURE 1:**
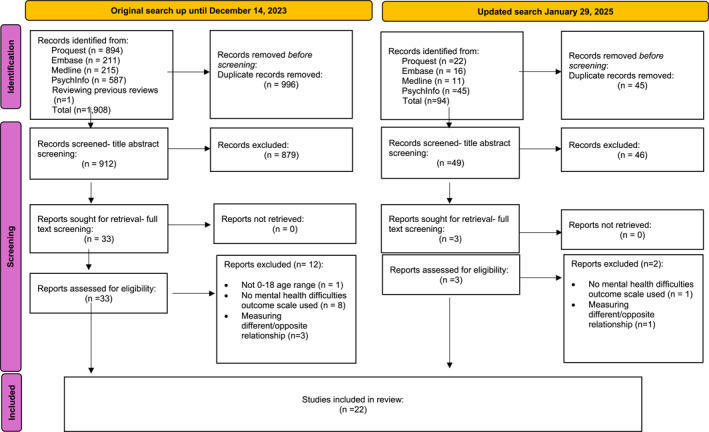
PRISMA flow diagram (adapted from Page et al., 2021).

The initial searches were completed on 14th December 2023. As shown in Figure 1, a total of 1908 papers were identified. Following de‐duplication, 912 unique references were screened at the title and abstract stage, followed by 33 full texts screened. A total of 21 studies met eligibility criteria. An updated search was completed on 29^th^ January 2025, where one further paper that met criteria for the review was identified and added.

### Study characteristics

Table 2 illustrates key information extracted from the included studies. The included 22 studies were published between 1988 and 2024: 11 were conducted within the United States, four in Australia, three in the United Kingdom, one in France, one in Belgium, one in The Netherlands and one in Jordan. Fifteen were a prospective cohort design and seven were cross sectional. The total number of participants across the 22 studies was 17,318 with an age range of 0–18 years. Out of the 22 studies included in the review, 19 studies measure mental health difficulties by including at least one carer or parent rated report using standardised measures such as Child Behaviour Checklist (CBCL; Achenbach, 2001). One study only used teacher report (Dumaret, 1988), one study used reports from social workers (Barber & Delfabbro, 2003), and one study used self‐report (Mishra et al., 2020).

**TABLE 2:** Study characteristics and placement operationalisation.

Author	Year	Region	Study design	*N*	Age range	MH measure	How placement instability was measured	How placement instability was defined	Time frame instability was measured
Aarons et al.,	2010	USA	Prospective cohort study	422	2–15	CBCL	Count (number of moves between waves of the study; between wave 1–3 and wave 3–4)	The child's physical location of residence had to change	3 years
Asif et al.,	2024	Australia	Prospective cohort study	4126	0–17	1) CBCL 2) BITSEA Combined to create a total socio‐emotional difficulties score	Count Measured as the distinct number of placement changes per 1000 care days	Number of distinct placement moves (to a different carer)	2.7 years
Barber and Delfabbro	2003	Australia	Prospective cohort study	235	4–17	CBCL	Coded into groups (3) 1) Stable (same placement) 2) Unstable (moved placement within both follow up periods) 3) Unstable‐stable‐ (changed placements between Baseline‐T2 but achieved a stable foster placement during T2‐T3)	Any change in placement	8 months
Beck	2006	UK	Cross sectional	786	3–18	SDQ	Count	No further details provided	1 year
Dumaret	1988	France	Cross sectional	157	7–15	Rutter B scale	Coded into groups (3) 1) 1 previous placement 2) 2 previous placements 3) 3 or more previous placements	Number of placements prior to admission to the children's village	Lifetime
Hiller et al.,	2023	UK	Prospective cohort study	672	2–16	SDQ	Count	Total number of placement providers over the first 3 years in care	3 years
Hiller and St Clair	2018	UK	Prospective cohort study	217	4–18	SDQ	Count	The total number of individual placement providers over the first 5 years in care (any length of placement (including respite care or temporary placements).	5 years
Hu et al.,	2024		Prospective cohort study	345	3–17	CBCL	Coded into groups (5) 1) 1 2) 2 3) 3 4) 4 5) 5 or more placements	The total number of placements experienced by a child.	Lifetime
Hussey and Guo	2005	USA	Cross sectional	119	4–18	Devereux scales of mental disorders (DSMD)	Count	Previous out of home placements prior to their current admission	Lifetime
Lewis et al.,	2007	USA	Cross sectional	102	5–6	CBCL	Count	Any placement with a new caregiver was counted as one placement.	Lifetime
Linares et al.,	2010	USA	Prospective cohort study	252	3‐ >12	Parent report: The computer‐based Diagnostic interview schedule for children (CDISC4) Teacher report: Sutter Eyberg student behaviour inventory‐revised	Count Time‐varying moves indicated the number of moves at each wave (0–7). Average moves coded as time invariant indicated the mean sum of moves across waves (0–7).	Number of foster home changes	4 years
MacKenzie et al.,	2014	Jordan	Cross sectional	134	1.5–12	CBCL	Count	Based on policy the care centres moved children at different time points. This was used to calculate approximate change clocks.	Lifetime
Mishra et al.,	2020	USA	Prospective cohort study	1657	9–17	Youth self report (YSR)	Coded into groups (6) *OOHP—out of home placement* 1) ‘OOH three times’ 2) ‘OOHP three times with one or more change in placement’ 3) ‘OOHP two times’ 4) ‘OOHP two times with change in placement’ 5) ‘OOHP one time’; and 6) ‘no OOHP’	Any change in out of home placement	3 years
Newton et al.,	2000	USA	Prospective cohort study	415	0–17	CBCL	Count	Every change in placement during the first 18 months after entry to care	1.5 years
Proctor et al.,	2010	USA	Prospective cohort study	279	6–14	CBCL	Coded into groups (2) 1) 1 (same caregiver since previous interview) 2) 0 (different caregiver since previous interview) Then used to create a continuous measure across all 5 time points ranging from (child was never living with the same primary caregiver) to 5 (child was living with the same primary caregiver across all 5 time points).	Caregiver stability rather than placement move, coded at each time point of the study.	8 years
Rosenthal and Villegas	2010	USA	Prospective cohort study	4080	0–16	CBCL	Coded into groups (3) 1) 0 (0 changes) 2) 1 (1 change) 3) 2 (2 or more changes)	Include move to foster homes (kin and non‐kin), group homes, residential treatment, and other placement settings.	8 years
Rubin et al.,	2008	USA	Perspective cohort study	1309	<2, 2–10, >10	CBCL	Coded into groups (3) 1) Early stable (children who achieved a long‐lasting placement within 45 days of entry into out‐of‐home care, which was maintained for the study period) 2) Late stable (achieved a long‐lasting placement, but only after 45 days, with a duration of at least half of the study period) 3) Unstable (children failed to achieve a long‐ lasting placement during study period)	No additional information provided.	3 years
Rubin et al.,	2007	USA	Perspective cohort study	729	0–15	1) CBCL 2) Temperament scores Combined to create total behaviour problems	Coded into groups (3) (same as above, Rubin et al., 2008).	No additional information provided.	3 years
Strijker et al.,	2008	Netherlands	Prospective cohort study	419	0–18	Behaviour problems questionnaire	Count	Current foster family placements are not included in establishing the placement history. A movement is described in this study as each transfer of a child to another placement without his parents.	1.5 years
Tarren‐Sweeney	2008	Australia	Cross sectional	347	4–11	ACC CBCL	Count	The number of temporary placements pre‐care and number of permanent placements in‐care	Pre‐care and in‐care
Vanschoonlandt et al.,	2012	Belgium	Cross sectional	186	3–18	CBCL	Count	Number of previous out of home placements	Lifetime
Villodas et al.,	2016	USA	Prospective cohort study	330	4–12	Caregiver report‐CBCL Youth self‐ report YSR	Count	Number of placements during the first 18 months in care (i.e., before permanent placement).	1.5 years

### Quality assessment

Table 3 provides a summary of the quality appraisal of the studies. Seven were rated as high quality, 14 studies were rated as moderate quality, one study was rated as poor quality, which was also the oldest paper (Dumaret, 1988) and may have followed different reporting guidance. All were considered eligible for inclusion in the descriptive and narrative synthesis (further detail regarding quality assessment can be found in Supporting Information S1: Appendix S1).

**TABLE 3:** Quality appraisal of studies using the CCAT (Crowe, 2013).

Author	Year	Preliminaries	Introduction	Design	Sampling	Data collection	Ethical matters	Results	Discussion	Total score/40	Total % score	Quality rating
Aarons et al.,	2010	4	5	3	4	3	3	4	5	31	78%	High
Asif et al.,	2024	4	5	3	3	3	1	4	4	27	68%	Moderate
Barber and Delfabbro	2003	4	4	2	3	4	0	4	3	24	60%	Moderate
Beck	2006	3	2	1	3	3	2	2	4	20	50%	Moderate
Dumaret	1988	2	2	2	3	2	0	3	3	17	43%	Poor
Hiller et al.,	2023	4	5	4	4	4	4	4	5	34	85%	High
Hiller and St. Clair	2018	4	3	3	4	4	4	4	4	30	75%	Moderate
Hu et al.,	2024	4	4	3	3	3	2	4	4	27	68%	Moderate
Hussey and Guo	2005	3	4	3	3	3	0	4	4	24	60%	Moderate
Lewis et al.,	2007	4	5	3	3	4	0	4	4	28	70%	Moderate
Linares et al.,	2010	4	3	4	4	4	1	4	4	28	70%	Moderate
MacKenzie et al.,	2014	4	4	4	4	4	5	4	4	33	83%	High
Mishra et al.,	2020	4	4	4	4	4	4	4	4	32	80%	High
Newton et al.,	2000	4	4	4	4	4	0	4	5	29	73%	Moderate
Proctor et al.,	2010	4	4	4	4	4	3	4	4	31	78%	High
Rosenthal and Villegas	2010	4	5	4	4	4	0	4	5	30	75%	Moderate
Rubin et al.,	2008	4	3	4	4	3	3	4	4	30	75%	Moderate
Rubin et al.,	2007	4	4	4	4	4	2	4	4	30	75%	Moderate
Strijker et al.,	2008	4	4	4	3	4	0	4	3	27	68%	Moderate
Tarren‐Sweeney	2008	5	4	4	4	3	2	4	5	31	78%	High
Vanschoonlandt et al.,	2012	4	4	3	3	4	0	3	4	25	63%	Moderate
Villodas et al.,	2016	5	5	4	4	4	3	4	4	32	80%	High

#### Measurement of placement instability or moves

Overall, there was variation in how studies measured and characterised placement instability. As you can see from Table 2, 13 studies measured placement instability by counting and reporting on number of moves and nine studies created groups to represent instability in placement. Grouping approaches varied, some labelled groups as stable or unstable (and variations of this) (Barber & Delfabbro, 2003; Rubin et al., 2007, 2008) or used numbers to code variations in instability (e.g., 0 = no changes, 1 = 1 change, 2 = 2 or more changes) (Dumaret, 1988; Rosenthal & Villegas, 2010).

The time frames in which studies calculated the number of placement moves also varied. Fifteen studies measured placement moves during their specific study period, which ranged from 8 months to 8 years. One study measured the number of moves since entry to foster care (Tarren‐Sweeney, 2008). Six studies did not explicitly state the time frames and inferred lifetime instability (Dumaret, 1988; Hu et al., 2024; Hussey & Guo, 2005; Lewis et al., 2007; MacKenzie et al., 2014; Vanchoonlandt et al., 2012).

#### Impact of placement in/stability on mental health outcomes

From reviewing the papers, the results were grouped and narratively synthesised based on mental health domain (total mental health difficulties, internalising or externalising; Supporting Information S1: Table S1 provides an overview of the main findings of each study). Where appropriate, these results were further explicated based on whether the analysis was adjusted or unadjusted for covariates, and how the outcome was measured (continuous or clinical cutoffs). The covariates that were used to organise the synthesis included initial mental health, sex, and age, as those were the three most consistently used across studies. Other covariates may have been included but those varied across studies and as such were not highlighted in the synthesis.

For a subset of these studies (see Supporting Information S1: Table S1 for reasons for exclusion), a meta‐analysis was conducted. To calculate the overall effect of placement instability on mental health outcomes two random effect meta‐analyses were conducted, one with externalising difficulties (*k* = 14) as the outcome and another with internalising difficulties (*k* = 11) as the outcome. After the initial analysis was conducted in both the externalising and internalising models, both models presented with high heterogeneity (I^2^ above 80%). One study (Villodas et al., 2016) was deemed an outlier. As such the models were re‐run without this study (overall conclusions remained the same). The final meta‐analyses included 13 papers for externalising difficulties and 10 for internalising difficulties reported on below.

#### Total mental health difficulties

Eight papers included in the review examined total mental health difficulties, with significant associations between instability and mental health difficulties noted in all eight studies (Beck, 2006; Hu et al., 2024; Hussey & Guo, 2005; Lewis et al., 2007; Newton et al., 2000; Rubin et al., 2008; Tarren‐Sweeney, 2008; Vanschoonlandt et al., 2012). Two papers were not eligible for the meta‐analysis (Beck, 2006; Hussey & Guo, 2005), and four papers also reported on internalising and externalising difficulties separately, therefore a meta‐analysis on total difficulties was not conducted as the effects of these studies would be captured in internalising and externalising difficulties' analyses. Two remaining papers only reported on total mental health difficulties (Hu et al., 2024; Tarren‐Sweeney, 2008), and these two papers were not included in the meta‐analysis.

#### Externalising difficulties

##### Narrative synthesis

Overall, 18 studies examined the effect of placement instability on externalising outcomes.

##### Studies adjusted for covariates

Eleven studies were adjusted for covariates, nine of which found a significant association between placement instability and externalising difficulties (Aarons et al., 2010; Hiller et al., 2023; Hussey & Guo, 2005; Linares et al., 2010; Mishra et al., 2020; Newton et al., 2000; Proctor et al., 2010; Rubin et al., 2007; Villodas et al., 2016). Proctor et al. (2010) found the significant effect in the stable positive adjustment profile but not in the increasing adjustment profile. Barber and Delfabbro (2003) found improvements in externalising difficulties (except for hyperactivity) for children in stable placement profiles but also found improvements for children in unstable placement profiles.

###### Adjusted for initial levels of mental health

Four of these studies found an effect when adjusting for child's initial levels of mental health (Aarons et al., 2010; Newton et al., 2000; Rubin et al., 2007; Villodas et al., 2016), whereas one study found no significant effect (Rosenthal & Villegas, 2010). This suggests that, for the most part, the association between placement instability and externalising difficulties was found while controlling for initial levels of mental health difficulties.

###### Adjusted for child age and sex

Five studies controlled for child age and sex (Hussey & Guo, 2005; Linares et al., 2010; Mishra et al., 2020; Newton et al., 2000; Rubin et al., 2007), and one controlled for child age (Hiller et al., 2023), all of which found significant associations between placement instability and externalising difficulties although Hiller et al., found this effect consistently for chronic but not delayed trajectory group. Taken together the results suggest that the association between placement instability and externalising difficulties was found across different age groups as well as sex. One study that adjusted for child age found no significant effect (Rosenthal & Villegas, 2010).

##### Unadjusted

The remaining seven studies were unadjusted. Five of these studies found an association between placement instability and externalising difficulties (Dumaret, 1988; Hiller & St. Clair, 2018; Lewis et al., 2007; Strijker et al., 2008; Vanschoonlandt et al., 2012). MacKenzie et al. (2014), did not find a significant association, and was also the only study to measure placement instability by using time since last move. It was unclear what significance testing was used in Beck (2006) but they reported that young people who move placement frequently were more likely to have a probable conduct disorder.

##### How outcome measure was coded

Eleven studies measured externalising difficulties on a continuous scale, two of which did not find significant associations (MacKenzie et al., 2014; Rosenthal & Villegas, 2010). Six measured externalising difficulties using clinical cutoffs, all of which found significant associations.

##### Meta‐analysis

**FIGURE 2:**
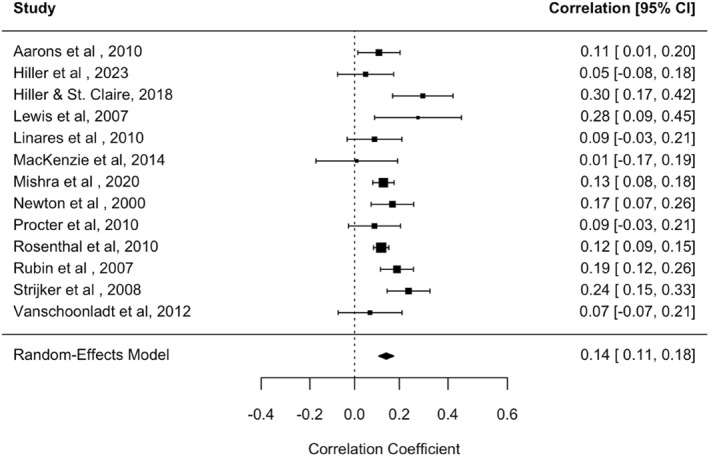
Forest plot for the random effects meta‐analysis of externalising difficulties.

A total of 13 studies were included in the meta‐analysis, with a total sample size of 8149.5. The degree of heterogeneity in the meta‐analysis was calculated using a restricted maximum‐likelihood estimator. The I^2^ found a 43.4% heterogeneity across studies and the Cochranes *Q* was significant (*p* = 0.03), suggesting small heterogeneity across studies. Based on the level of heterogeneity, we can assume that just over half of variation is a result of differences in true effect size. To assess for publication bias, the funnel plot was visually examined and looked approximately symmetrical, suggesting no evidence of publication bias. Egger's regression and the Rank correlation test were also examined. Neither of these tests were statistically significant suggesting no evidence of publication bias. The meta‐analytical results for the association between placement instability and externalising difficulties revealed a combined correlation of *r =* 0.14, 95% CI = 0.11, 0.18, which represents a small but significant effect (*p* < 0.001). This is visualised in the Forest plot below (Figure 2).

#### Internalising difficulties

##### Narrative synthesis

Overall, 14 studies examined the effect of placement instability on internalising outcomes.

##### Studies adjusted for covariates

Ten were adjusted for covariates, eight of which found significant effects of placement instability on internalising difficulties (Asif et al., 2024; Hiller et al., 2023; Hussey & Guo, 2005; Mishra et al., 2020; Newton et al., 2000; Proctor et al., 2010; Rosenthal & Villegas, 2010). Proctor et al. (2010) found the significant effect in the stable positive adjustment profile but not in the increasing adjustment profile. Barber and Delfabbro (2003) found that children in stable placement profiles showed a steady trend towards improvement in internalising difficulties, however, they also found that children in unstable placement profiles also showed an improvement. Rosenthal and Villegas (2010) found that placement change predicted internalising difficulties at all follow ups except for the last follow up.

###### Adjusted for initial levels of mental health

Four studies had controlled for initial levels of mental health (Aarons et al., 2010; Newton et al., 2000; Rosenthal & Villegas, 2010; Villodas et al., 2016). Two found a significant effect of placement instability on a change in internalising difficulties, and one found no significant effect (Aarons et al., 2010). Villodas et al. (2016) found that placement instability was only significantly associated with changes in youth self‐report but not carer report.

###### Adjusted for child age and sex

Of the six studies that adjusted for child age and sex, three adjusted for child age and sex (Hussey & Guo, 2005; Mishra et al., 2020; Newton et al., 2000), two adjusted for child age (Hiller et al., 2023; Rosenthal & Villegas, 2010) and one study adjusted for sex (Asif et al., 2024). All of the studies reported on significant associations, although Rosenthal and Villegas (2010) did not find significant effects with changes in the last follow up and Hiller et al. (2023) did not find this effect for the delayed group. This suggests that the association between placement instability and internalising difficulties was present irrespective of age or sex.

##### Unadjusted

Four studies were unadjusted, three of which found a significant effect (Hiller & Clair, 2018; MacKenzie et al., 2014; Vanschoonlandt et al., 2012), whereas only Lewis et al. (2007) did not find a significant association.

##### How outcome measure was coded

Eight studies measured internalising difficulties using a continuous scale score, two of which did not find significant effects (Aarons et al., 2010; Lewis et al., 2007). Six studies used clinical cut offs, all finding significant effects.

##### Meta‐analysis

**FIGURE 3:**
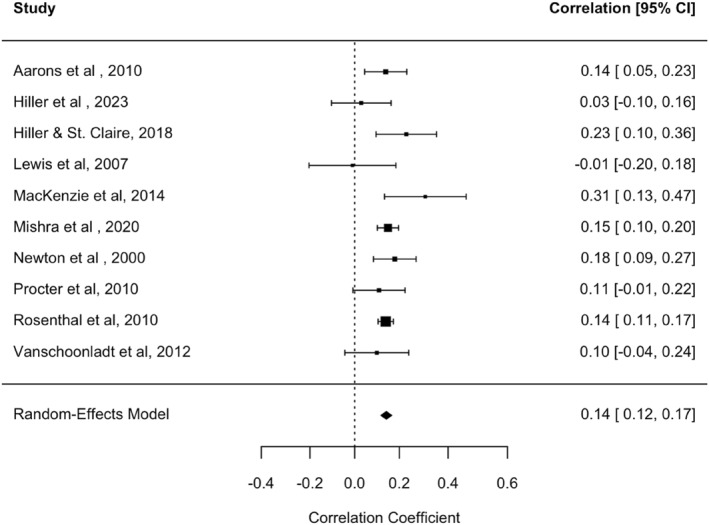
Forest plot for the random effects meta‐analysis for internalising difficulties.

A total of 10 studies were included in the meta‐analysis, including 6771 participants. The I^2^ found a 16% heterogeneity across studies and the Cochranes *Q* was not significant (*p* = 0.24), suggesting low heterogeneity across studies. To assess for publication bias, the funnel plot was visually examined and looked approximately symmetrical, suggesting no evidence of publication bias. Egger's regression and the Rank correlation test were also not statistically significant suggesting no evidence of publication bias. The meta‐analytical results for the association between placement instability and internalising difficulties revealed a combined correlation of *r =* 0.14, 95% CI = 0.12, 0.17, which represents a small but significant effect (*p* < 0.001). This is visualised in the Forest plot below (Figure 3).

## DISCUSSION

The main aim of this study was to examine the association between placement instability and internalising and externalising mental health outcomes of CECYP. A secondary aim was to explore how placement instability was measured in those studies to better understand (in)consistencies in the literature and make recommendations for future work. Based on narrative synthesis and meta‐analysis of observational data, there was a small but significant association between placement instability and both internalising and externalising difficulties. Importantly, this effect was in some cases independent of the child's initial levels of mental health and in many cases independent of the child's age and sex.

This study offers evidence supporting the finding that greater placement instability is significantly associated with worse mental health outcomes, across samples with a wide age range from early childhood to late adolescence. This means that frequent moves between various types of care placements have a negative impact on CECYP. Services should aim to provide stable placements, allowing CECYP to develop a sense of stability and consistency in both their physical environment and also with their caregivers, in order to protect and promote their overall wellbeing (Lockwood et al., 2015) and prevent mental health difficulties. Although the pooled effects from the meta‐analyses were small, many were based on adjusted associations controlling for initial mental health difficulties, age, sex, and other confounders and therefore small effects are expected. Recent UK focussed research that has examined bivariate associations has shown stronger effects, with children and young people being twice as more likely to have mental health difficulties as a function of placement instability (Varnish et al., 2025). Nonetheless, small effects in psychological and mental health research can be impactful, especially when applied to a population where you can see disproportionate effects for those most at risk (i.e., high end of the distribution) (Carey et al., 2023).

This effect was found across studies that used various operationalisations of placement instability, with the most utilised metric being count or frequency of moves. Even when this was used, timeframes for capturing instability varied depending on available records as well as what constituted a placement move also varied depending on whether all moves counted or only when one is moved from birth family's care. Some studies also categorised level of stability or used profiles to understand stability and instability in placements over time. This variability across studies makes it difficult to ascertain what constitutes a level of change in placement instability and to what extent it has an impact on outcomes. Count seems like the most consistent approach to capturing number of moves between care environments, but the parameters of what constitutes a move should be clarified by individual studies moving forwards. Future analysis regarding the dimensions of placement instability, how to measure them, and their unique impacts on outcomes will benefit applied work and social care policy generally.

It is important to note here that there can be a difference between promoting stability in a placement and seeking permanence, which is the imposition of a legal order which imbues the new carers with legal rights over the child and prevents the child from moving on without the application of a new legal order (Atkinson et al., 2023). With permanence comes the need to balance the rights of parents in offering a realistic prospect of making changes to have their children live with them (e.g., Article 8, European Convention on Human Rights (Council of Europe, 1950), Article 9 UNCRC) with the rights of children who, if they cannot reside with their birth parents, have the right to care which is continuous (Article 20, UNCRC). Guidelines for the Alternative Care of Children (United Nations, 2009), endorsed by the general assembly, emphasise the importance of permanency planning and the need to prioritise family‐based care over institutional care. This tension can be difficult to navigate and given that all forms of care, in the absence of a legal deliberation of permanence, are by their very nature temporary, and thus the possibility of placement disruption is built into the process (Department of Education, 2013). This review demonstrates the impact of instability in placements on children while in care, which is distinct from factors or outcomes of being placed in a permanent placement (e.g., Akin, 2011; Biehal et al., 2019; Neil et al., 2019).

We already know that mental health difficulties, specifically externalising difficulties, predict placement instability or disruption for CECYP (Konijn et al., 2019). Together with our findings from this review, this highlights the cyclical relationship between mental health difficulties and placement instability. That is, CECYP generally experience high mental health difficulties (Engler et al., 2022), these difficulties may be deemed challenging to manage, particularly in the case of externalising difficulties. These difficulties could then lead to a child being moved, which in turn further impacts negatively on their mental health, both increasing risk of internalising and externalising difficulties as shown in this study, risking leading to further placement breakdown and instability. This cycle of mental health difficulties and placement breakdown risks exacerbating difficulties for a highly vulnerable population group if left unmanaged and unsupported. Findings from a qualitative study with carers suggest that ‘things might get worse before they get better’, implying that moving children when they display difficult behaviour while in care might be inappropriate and that recovery from maltreatment takes time and adjustment to new care placements (Turner et al., 2022). This then means that rather than moving children or young people at early signs of mental health difficulties, we should prioritise providing support for stability in caring, relationships, and mental health. This also highlights a need for effective intervention for carers and the right resourcing for placements, to ensure that the systems surrounding CECYP are well equipped to support their mental health needs and difficulties when they arise, to mitigate the negative sequelae of placement instability.

Importantly, the journey for CECYP continues into adulthood, with care experience being a risk factor for negative health outcomes in care leavers (those who transition out of care into adulthood) regardless of placement type, whereas placement stability while in care being associated with better mental health and wellbeing in care leavers (Power & Hardy, 2024). Power and Hardy note the need for further studies to discern nuances in these relationships by looking at potential moderators, variations in care systems, and experiences pre and post care are necessary. Nonetheless, these findings further highlight the importance of considering placement instability for both short term (while in care) and long‐term outcomes of young people once they have left care.

### Strengths, limitations and future directions

A strength of this review is the large number of prospective cohort studies included, which captures the impact of placement instability over time. However, there are limitations to a meta‐analysis of observational data, which helps us understand associations but does not capture causation. Therefore, it is important to consider confounding variables and potential bias that may also be influencing the association between placement instability and mental health outcomes. Most CECYP will have been exposed to some level of adversity, which was not captured in the current model. Research examining exposure to Adverse Childhood Experiences (ACEs) on mental health outcomes finds that the more ACEs experienced by a child the more likely they are to report a mental health difficulty (Hinojosa & Hinojosa, 2024). Further research could explore these relationships and how those interact with placement instability (e.g., whether severity of ACEs sensitises CECYP to effects of placement instability). In addition, because we included adjusted effects in the meta‐analysis, this introduces variation and bias due to different confounders adjusted for across studies. Future work might want to prioritise synthesising bivariate associations or include subsets that have controlled for the same set of confounders to mitigate bias and variation.

As highlighted earlier, there was large variation in how the different studies measure placement instability, or what constitutes a change in placement, which could in part explain some of the heterogeneity found in the meta‐analysis. Some of the categorisation of instability may also miss out on variation in terms of number of moves experienced and be less sensitive to estimating effects on mental health outcomes. This highlights the need for a consensus within research on how placement instability is operationalised to assist future synthesis of the findings. There may be multiple dimensions to placement instability that would be of importance (e.g., duration, number of moves, number of carers, types of placements, etc.). Although out with the scope of this paper, it will be important to examine the moderating effect of placement type in future research. In addition, the time frames in which placement instability is measured varied across studies. Future research can assess whether variation in time frame impacts on the association between placement instability and mental health outcomes. Further, across the studies included in the review, the mental health outcomes are mostly measured by carers. This highlights the need for more research that includes youth report, especially in the internalising domain as youth are the preferred informant on their internalising difficulties (Caqueo‐Urízar et al., 2022).

### Conclusion

This review has evidenced that above and beyond many other factors (such as initial mental health, age, and sex), placement instability has a negative, albeit small, effect on mental health difficulties of CECYP. Providing stable and secure placements should be a priority in order to ensure the best outcomes for CECYP. As a population group, CECYP have been exposed to much adversity in their lives, and placement instability can further exacerbate these difficulties. A policy drive towards placement stability has been embraced in some jurisdictions, for example, by the Adoption and Safe Families Act (Adoption and Safe Families Act, 1997) in the US which determines strict timescales within which decisions about permanent placements must be made, while in other jurisdictions no such policy drivers exist (Whincup et al., 2018). It is imperative that policy and service providers ensure support for carers is available, to prevent the cycle of placement instability and meet the needs of CECYP.

## AUTHOR CONTRIBUTIONS

**Rosa Sparks**: Conceptualization; data curation; formal analysis; methodology; writing—original draft. **Gary Kainth**: Conceptualization; writing—review and editing. **Helen Minnis**: Conceptualization; supervision; writing—review and editing. **Jala Rizeq**: Conceptualization; formal analysis; supervision; writing—review and editing.

## CONFLICT OF INTEREST STATEMENT

The authors declare no conflicts of interest.

## ETHICAL CONSIDERATIONS

No primary data were collected as part of this review. Therefore, informed consent and ethics approval are not applicable for this review.

## Supporting information

Supporting Information S1

## Data Availability

Data sharing not applicable to this article as no new data were generated for this review.
