# Supplementary material for: A systematic review and meta‐analysis examining the impact of placement instability on the mental health outcomes of care experienced children and young people
Source: JCPP Adv. 2026 Jan 31;6(3):e70099. doi: 10.1002/jcv2.70099 (PMC13339400; doi:10.1002/jcv2.70099)
Supplement: Supplementary file 1 [file JCV2-6-e70099-s001.docx]

**A systematic review and meta-analysis examining the impact of placement instability on the mental health outcomes of care experienced children and young people**

**Supporting Information**

**Appendix S1. Additional quality appraisal information.**

Overall, the introduction and discussion sections of the papers in this review were of good quality, with clear aims and rationale for their research and practical implications of the research appropriately highlighted. Most studies administered widely used outcome measures to assess mental health outcomes (e.g. CBCL; SDQ); although commonly used with CECYP, some may not have been validated with this population. Nonetheless, there were some methodological limitations present. Most studies did not explicitly state the design of the study, or the justification for choosing that design or sampling method. None of the studies reported completing a-priori sample size calculations. Eight of the studies made no reference to ethical considerations and a further five studies made no mention to ethical approval.

**Table S1**

*Main Findings of the Studies (and associated effect sizes)*

| Author | Year | n  (=number included in the analysis) | Mental health domain (cut offs for grouping or continuous score) | Main finding | Adjusted or unadjusted |
| --- | --- | --- | --- | --- | --- |
| Aarons et al., | 2010 | 422 | Internalising and externalising (continuous score) | Placement changes between Waves 1-3 (baseline to 18 months) predicted more externalising behaviour problems at Wave 3 (β =.147, p <.01) but not internalising problems (β =.147, p >.05). Placement changes between Waves 3-4 (18 months- 36 months did not significantly predict either externalising or internalising difficulties behaviour problems at Wave 4 (β =.045, p >.05, β =.093, p >.05). | Adjusted |
| Asif et al., * | 2024 | 1300 | Internalising (cut off) | Placement stability (whilst controlling for covariates) had a significant association with socio-emotional development The probability of being in the typical range decreases over time as the number of placements increases. | Adjusted |
| Barber and Delfabbro* | 2003 | 120 | Externalising and Internalising (conduct, hyperactivity and emotionality) (continuous score) | Children in the stable placement group had a linear improvement in behaviour. Children in the unstable group displayed improvements (except in hyperactivity). Children in the unstable-stable group only showed improvement when their placement was unstable. | Adjusted |
| Beck * | 2006 | 109 young people and 162 carers | Total (likelihood of any psychiatric diagnosis) and externalising (conduct)(cut off) | Young people who move placement frequently were three times more likely than others to have any psychiatric diagnosis and to have a ‘probable’ conduct disorder. The numbers were too small to compare hyperactivity and emotional disorders. | Unadjusted |
| Dumaret * | 1988 | 92 | Externalising (continuous score) | The greater the number of placements experienced by the child led to a higher average score on the measure of behaviour problems. | Unadjusted |
| Hiller et al., | 2023 | Internalising n = 213  Conduct n = 228 hyperactivity n = 253 | internalising and externalising (conduct and hyperactivity) (cut offs) | Higher number of placement moves over the first three years in care predicted the higher likelihood of children and young people being in the chronic trajectory group (conduct OR= 1.38, 95% CI=1.18,1.60), hyperactivity (OR=1.14, 95% CI= 1.01,1.28), emotional (OR=1.17, 95% CI= 1.04, 1.32) as compared to the resilient group. Number of placement moves over the first three years in care did not significantly predict the likelihood of children being in the delayed trajectory group in the internalising or conduct problems but did for hyperactivity as compared to the resilient group. | Adjusted |
| Hiller and St. Clair | 2018 | 207 | internalising and externalising (conduct and hyperactivity) at latest follow up (Year 5) (cut offs) | Main bivariate associations between placement moves and SDQ scores at latest follow up (year 5) suggest a positive association: for emotional problems (r=0.23, p<.05); for externalising difficulties these were separated across SDQ subscales for conduct (r=0.37, p<.01) and hyperactivity (r=0.22, p<.01). In addition, higher placement moves were associated with higher likelihood with being in the chronic trajectory on all subscales – chronic trajectories in terms of emotional and behavioural symptoms rated as abnormal initially and remaining the same throughout. | Unadjusted |
| Hu et al.,* | 2024 | 345 | Total problem (continuous score) | The proportion of children in the clinical trajectory group (those who had a persistently high mean total difficulties score over time) increased with higher number of placements at baseline, with 21.4 % of children who had experienced one placement to 42.1 % who had experienced 4 placements and 35.8 % of children who had experienced ≥5 placements. | Unadjusted |
| Hussey and Guo* | 2005 | 97 | Internalising, externalising and total problem (continuous score) | The number of previous out of home placements was positively associated with increased levels of psychiatric symptomatology across internalising difficulties (B= 0.95, p<.05), externalising difficulties (B=1.12, p<.01) and total difficulties (B=1.10, p<.01). | Adjusted |
| Lewis et al., | 2007 | 102 | Internalising, externalising and total problem (continuous score) | Placement instability was positively associated with externalising behavioural difficulties (r= .28, p<.05) and total difficulties (r=.21, p<.01). No significant associations were found between placement instability and internalising difficulties (r=-.01, p > .05). | Unadjusted |
| Linares et al., | 2010 | 252 | Externalising (inattention, hyperactivity) (continuous score) | Higher foster parent reports of hyperactivity were associated with higher number of foster home moves (β =.23, p =.043). No significant associations were found between number of placements and foster parent reports of symptoms of inattention (β =.07, p =0.578). Similarly, no effects were found for the birth parents reports of inattention or hyperactivity (β =.15, p =.0169; β =.00, p =.937) or teacher reports of inattention or hyperactivity (β =-.06, p =.729; β =-.14, p =.364). | Adjusted |
| MacKenzie et al., | 2014 | 115 | Internalising and externalising (continuous score) | Internalising difficulties were associated with stability in placement, as they decreased with time in placement (r= −.31, p<.001). However, externalising behavioural difficulties were not associated with stability (r = −.01, p > .05). | Unadjusted |
| Mishra et al., | 2020 | 1657 | Internalising(depression) and externalising (delinquency and aggression) (continuous score) | Results for out of home at all three timepoints with one or more placement change as compared to those who were never out of home: The higher number of placement changes were associated with negative outcomes for both internalising difficulties (B= 2.45, β = 0.10, p<.001) and externalising difficulties, across both delinquency (B=1.23, β = 0.09, p=.002) and aggression (B=2.19, β = 0.07, p=.021). | Adjusted |
| Newton et al., | 2000 | 415 | Internalising, externalising and total problem (continuous score) | Children who are exposed to higher number of placement changes are at greater risk of developing emotional and behavioural difficulties, for internalising (β =.125, p <.01), externalising (β =.122, p <.01) and total difficulties (β = .101, p<.01). | Adjusted |
| Proctor et al., | 2010 | 279 | Internalising and externalising (cut offs) | Stable positive adjustment in internalising (OR= 1.44, p<.01) and externalising symptoms (OR= 1.47, p<.05) was associated with carer stability. Those on increasing adjustment profiles (children who experienced high levels of initial behaviour difficulty but show increase in positive adjustment over time) were not significantly associated with carer stability for internalising (OR=1.57, p>.05) and externalising (OR=1.32, p> .05). | Adjusted |
| Rosenthal and Villegas | 2010 | 4080  Wave 4 to wave 5 n = 3175 (used in meta analysis) | Internalising and externalising (continuous score) | Placement change predicted internalising behavioural difficulties across 8 years (wave 1 to wave 3= (β =.094, p<0.05), wave 3 to wave 4 (β = .117, p<0.05) however not for the final wave (4 to wave 5) (β = .092, p> .05). They found no significant effect of placement change on externalising behaviours across all waves of the study, wave 1 to wave 3 (β = .061, p> .05) wave 3 to wave 4 (β = .001, p> .05) wave 4 to wave 5 (β = .067, p> .05) | Adjusted |
| Rubin et al.,* | 2008 | 1309 | Total problems (cut offs) | Children with unstable placement histories had a 49% probability of total mental health difficulties, compared to 32% probability if children had early stability in placements. They also found that children in kinship care were less likely to have unstable placements and were at a lower risk of difficulties at baseline. | Adjusted |
| Rubin et al., | 2007 | 689 | Externalising (cut offs) | Children in unstable placements were more likely to have behaviour problems than children who achieved early stability (OR=1.99). Children in foster care face placement instability independent of their problems and this instability has a significant impact on their behavioural well-being. | Adjusted |
| Strijker et al., | 2008 | Baseline n = 415 T2 n = 410 (used in meta analysis) | Externalising (continuous score) | Significant associations were found between the number of placements and severity of behavioural problems at baseline (*r*= .23, p=.0005) and at 1.5 years into placement (*r* = .24, p=.0005). | Unadjusted |
| Tarren-Sweeney* | 2008 | 347 | Total problems (continuous score) | Number of temporary placements prior to care were associated with total ACC (r = 0.15, p = .005) and number if placements in care were associated with CBCL (r = 0.15, p = .006) and ACC (r = 0.16, p = .003). | Unadjusted |
| Vanschoonlandt et al., | 2012 | 186 | Internalising, externalising and total problem (cut offs) | Number of previous placements predicted difficulties across both internalising (OR= 1.42, p=.01) externalising (OR= 1.29 p=.04) and total problems (OR = 1.34 p=.02). They found placement stability to be more important in predicting outcomes than type of placement (e.g. foster care or kinship care). | Unadjusted |
| Villodas et al., | 2016 | 330 | Internalising and externalising (cut offs) | Children in unstable placement trajectories had significantly poorer behavioural wellbeing than children in stable trajectories (only to stable group was used). For externalising difficulties this was found for both caregiver report (OR = 4.71, p = .004) and for youth self-report (OR = 5.15, p = .04). No significant associations were found for caregiver report of internalising difficulties (no statistics reported) but they were for youth self-report when comparing to stable placements (OR = 6.73, p = .02). | Adjusted |

*Note* * indicates studies not included in the meta-analyses for internalising and externalising difficulties. Reasons for exclusion; not reporting on type statistical analysis used (Beck, 2006; Dumaret, 1988, Rubin et al., 2008), type of effect size could not be transformed (Asif et al., 2024; Barber and Delfabbro, 2003), standardised effect size could not be calculated (Hussey and Guo, 2005), only reported effect size for total difficulties (Tarren-Sweeney, 2008; Hu et al., 2024).
